# Supplementary material for: Tumor Suppressor Properties of Small C-Terminal Domain Phosphatases in Clear Cell Renal Cell Carcinoma
Source: Int J Mol Sci. 2023 Aug 19;24(16):12986. doi: 10.3390/ijms241612986 (PMC10455398; doi:10.3390/ijms241612986)
Supplement: Supplementary file 1 [file ijms-24-12986-s001.zip › ijms-2546242-supplementary.pdf]

Supplementary Materials

Supplementary Table S1–S5

Table S1. Differential expression of SCP members in ccRCC (TCGA data, KIRC dataset).

(**A**)

Pooled samples

	Log2FC	FDR	*P*-value	avg. tumor CPM	avg. norm CPM	rel. LogCPM [normal]	pseudo Log2FC [T/N]
CTDSP1	0.3	$3×10^{-8}$	$1.2×10^{-8}$	211.9	172.7		
CTDSP2	-0.2	$7×10^{-5}$	$6×10^{-5}$	264.7	298.2		
CTDSPL	-1.4	$4×10^{-34}$	$7×10^{-35}$	61.2	157.8		
RB1	0.3	$9×10^{-8}$	$6×10^{-8}$	108.0	85.5		

(**B**)

Paired samples

	Log2FC (mean)	FDR	*P*-value	avg. tumor CPM	avg. norm CPM	Log2FC profile
CTDSP1	0.2	0.0001	$8×10^{-5}$	204.5	172.7	
CTDSP2	-0.1	0.29	0.29	286.1	298.2	
CTDSPL	-1.4	$2×10^{-10}$	$4×10^{-11}$	63.1	157.8	
RB1	0.5	$8×10^{-9}$	$3×10^{-9}$	124.0	85.5	

Upper plot (**A**) demonstrates the comparison of two pools of samples (tumors versus normal tissues); lower plot (**B**) – each tumor was compared to the adjacent normal tissue. Log2FC – binary logarithm of fold change; CPM – read counts per million. Mini-plots in right cells (**A**) show the relative binary logarithm of CPM values normalized to the average value across all normal samples, or Log2FC for paired samples (**B**). Red/blue – the expression is above/below the average value for normal samples (pooled test, **A**) or up-/down-regulated for a current patient (paired samples, **B**). The vertical axis scale is from −4 to +4 (from 16-fold down-regulation to 16-fold up-regulation).

Table S2. Co-expression of SCP family phosphatases and their target RB1 in clear cell renal carcinoma (TCGA data).

	CTDSP1	*RB1*	*CTDSP2*	*CTDSPL*	avg. LogFC	LogFC profile
CTDSP1		-0.07	-0.15	-0.21	0.3	
RB1	0.08		0.21	0.00	0.3	
CTDSP2	**$3x10^{-4}$**	**$1.3x10^{-7}$**		0.46	-0.2	
CTDSPL	**$1.4x10^{-7}$**	0.9	**$2x10^{-33}$**		-1.4	

corr. coeff.

p-values

Spearman's rank correlation coefficient r_s is shown in the upper part of correlation matrix, *p*-values – in the lower part.

Table S3. Methylation profiling of *CTDSPL* (**A**) and *CTDSP2* (**B**) genes in ccRCC (TCGA data, KIRC dataset, 320 tumors and 160 normal samples).

(**A**)

CTDSPL

Chromating state (ENCODE)								Beta-value median					correlations with RNA-Seq			
H1-hESC	K562	GM12878	HepG2	HUVEC	HeLa-S3	position (chr3)	CpG ID	T	N	delta beta-value (Tumor minus Normal), paired samples	HM score [pooled]	HM score [paired]	r [pooled]	p	r [paired]	p
TSS	TSS	T	TSS	E	TSS	37 902 230	cg00056624	0.02	0.02		3.9	0.2	-0.08	0.137	-0.01	0.952
TSS		R	TSS	E	E	37 902 414	cg27201297	0.03	0.03		3.7	0.3	-0.10	0.053	0.17	0.416
E		R	TSS	E	E	37 902 565	cg03637781	0.04	0.03		5.8	0.5	-0.26	7×10^{-7}	-0.15	0.472
E		R	TSS	E	E	37 902 571	cg02843755	0.05	0.04		5.3	0.3	-0.20	0.0002	-0.29	0.162
TSS		R	TSS		E	37 902 742	cg12824246	0.09	0.07		16.8	2.0	-0.23	1×10^{-5}	0.46	0.023
	E		TSS	E		37 903 683	cg11239633	0.07	0.07		4.5	0.1	-0.09	0.1063	-0.17	0.418
	E		TSS	E		37 903 685	cg11171719	0.05	0.05		2.4	0.0	-0.09	0.1049	-0.10	0.645
TSS	TSS	R	TSS	E	E	37 904 035	cg01439112	0.18	0.16		15.6	1.8	-0.44	7×10^{-18}	-0.29	0.175
TSS	E	R	TSS	E	E	37 904 294	cg12919119	0.36	0.26		37.7	8.0	-0.57	4×10^{-31}	-0.56	0.004
TSS	E	R	TSS	E	E	37 904 476	cg08229360	0.21	0.16		25.2	3.8	-0.49	8×10^{-22}	-0.50	0.014
	CTC	CTCF		CTC	CTC	37 906 587	cg12386061	0.13	0.26		0.0	0.0	-0.01	0.8394	-0.51	0.011
R	R	R	T	T	R	37 920 131	cg22380476	0.90	0.89		15.7	2.6	0.23	1×10^{-5}	0.13	0.542
R		R	T	T	T	37 926 308	cg13430960	0.92	0.80		130.9	360.4	-0.48	1×10^{-20}	-0.15	0.481
R		R	E	T	R	37 944 839	cg13333722	0.94	0.67		204.5	934.8	-0.64	1×10^{-41}	-0.33	0.118
R	R	R	T	R	R	37 959 357	cg08545213	0.93	0.84		121.7	280.2	-0.40	7×10^{-15}	0.25	0.241
R	R	R		R	R	37 965 819	cg05990080	0.76	0.73		42.2	16.1	-0.28	1×10^{-7}	-0.23	0.279
	R	R		R	T	37 966 888	cg12605080	0.83	0.72		83.8	75.5	-0.44	8×10^{-18}	-0.27	0.199
R	R	R	TSS	E		37 977 734	cg02355558	0.53	0.57		2.3	0.0	-0.19	0.0003	-0.44	0.03
WE	R	R	T	E	E	37 987 839	cg12902896	0.24	0.21		27.3	7.4	-0.31	4×10^{-9}	-0.39	0.06
WE	R	R	T	WE	R	38 003 248	cg01534527	0.46	0.36		55.8	62.3	-0.42	1×10^{-15}	-0.41	0.047
R	R	R	T		R	38 009 912	cg26160492	0.95	0.97		0.0	0.0	-0.13	0.0136	0.04	0.845
R	R	R	T		R	38 009 935	cg04787317	0.90	0.79		112.5	276.1	-0.56	2×10^{-29}	-0.20	0.353
T	R	R	T		R	38 010 392	cg08171483	0.76	0.63		82.6	102.2	-0.40	2×10^{-14}	-0.20	0.349
R	R	R	T		T	38 010 716	cg26054057	0.61	0.49		59.0	29.5	-0.45	2×10^{-18}	-0.32	0.123
T		R	T	T	T	38 010 839	cg07816047	0.43	0.44		16.4	0.8	-0.28	1×10^{-7}	-0.35	0.093
T		R	T	T	T	38 010 903	cg15555217	0.71	0.79		0.0	0.0	-0.15	0.0092	-0.13	0.635

(**B**)

CTDSP2

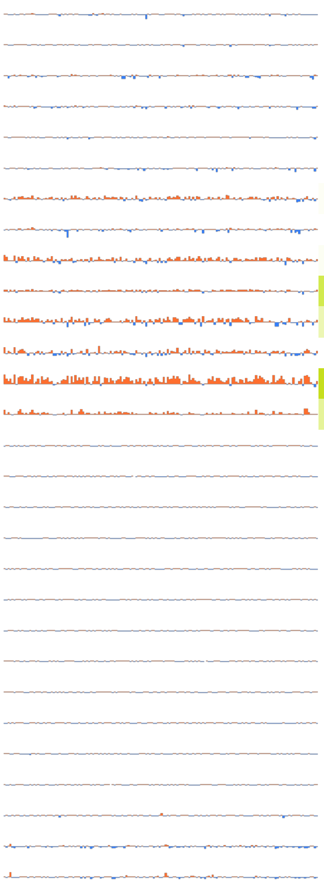

Chromating state (ENCODE): H1-hESC	K562	GM12878	HepG2	HUVEC	HeLa-S3	position (chr12)	CpG ID	Beta-value median: T	N	delta beta-value (Tumor minus Normal), paired samples	HM score [pooled]	HM score [paired]	correlations with RNA-Seq: r [pooled]	p	r [paired]	p
T	T	T	T		T	58 217 367	cg07246187	0.83	0.84		0.0	0.0	-0.05	0.3577	-0.29	0.174
T	T	T	T	T	T	58 218 060	cg04118006	0.93	0.93		2.9	4.3	0.00	0.9346	-0.42	0.041
T	T	T	T	T	T	58 218 603	cg07617764	0.85	0.83		29.8	7.6	-0.04	0.4517	-0.02	0.912
T	T	T	T	T	T	58 218 621	cg01190168	0.85	0.84		0.2	0.0	-0.04	0.4681	-0.18	0.413
T	T	T	T	T	T	58 218 673	cg15851964	0.92	0.92		0.0	3.4	0.03	0.5838	0.01	0.968
T	T	T	T	T	T	58 219 113	cg17169243	0.77	0.77		0.0	0.2	0.09	0.0889	0.09	0.683
T	T	T	T	T	T	58 219 818	cg09263904	0.68	0.64		43.9	23.7	-0.22	5×10^{-5}	-0.36	0.082
T	T	T	T	T	T	58 224 503	cg21149967	0.87	0.85		30.9	12.5	-0.14	0.0091	0.01	0.979
T	T	TSS	T	E	T	58 232 985	cg02566627	0.49	0.36		44.4	22.5	-0.18	0.0009	-0.17	0.432
TSS	T	TSS		T	T	58 236 349	cg03228931	0.91	0.82		102.8	206.8	-0.24	1×10^{-5}	-0.26	0.226
TSS	T	TSS			T	58 236 713	cg01644731	0.72	0.58		66.9	71.2	-0.25	4×10^{-6}	-0.19	0.379
	T	TSS	PF	T	T	58 237 273	cg16915316	0.36	0.30		26.3	14.4	-0.25	3×10^{-6}	-0.41	0.044
WE	T	TSS		T	WE	58 237 707	cg04415672	0.45	0.20		133.6	676.5	-0.33	3×10^{-10}	-0.27	0.21
TSS		TSS				58 238 233	cg09183316	0.11	0.04		76.3	21.8	-0.32	1×10^{-9}	-0.51	0.01
TSS	TSS	TSS	TSS	TSS	TSS	58 239 135	cg03091738	0.03	0.02		1.7	0.0	0.00	0.9762	-0.06	0.793
TSS	TSS	TSS	TSS	TSS	TSS	58 239 325	cg05902531	0.01	0.01		0.8	0.0	0.04	0.4433	0.02	0.944
TSS	TSS	TSS	TSS	TSS	TSS	58 239 493	cg26639561	0.04	0.04		2.4	0.0	0.02	0.6587	-0.20	0.361
TSS	TSS	TSS	TSS	TSS	TSS	58 239 513	cg02540427	0.04	0.04		2.1	0.0	0.12	0.0214	-0.08	0.705
TSS	TSS	TSS	TSS	TSS	TSS	58 239 953	cg13667676	0.01	0.01		1.4	0.0	0.02	0.6549	-0.29	0.168
TSS	TSS	TSS	TSS	TSS	TSS	58 240 177	cg11762629	0.03	0.03		2.6	0.0	-0.04	0.4439	-0.17	0.42
TSS	TSS	TSS	TSS	TSS	TSS	58 240 443	cg12592194	0.03	0.03		2.5	0.0	0.03	0.6143	0.01	0.946
TSS	TSS	TSS	TSS	TSS	TSS	58 240 788	cg20499859	0.02	0.02		1.2	0.0	-0.08	0.1643	-0.15	0.475
TSS	TSS	TSS	TSS	TSS	TSS	58 240 810	cg19593762	0.02	0.01		2.4	0.1	-0.10	0.0729	-0.23	0.274
TSS	TSS	TSS	TSS	TSS	TSS	58 240 818	cg26645635	0.01	0.01		1.5	0.0	-0.05	0.3226	-0.17	0.423
TSS	TSS	TSS	TSS	TSS	TSS	58 240 844	cg05342250	0.03	0.03		2.0	0.0	0.10	0.0702	-0.11	0.6
TSS	TSS	TSS	TSS	TSS	TSS	58 240 897	cg16444117	0.01	0.01		1.4	0.0	-0.11	0.0401	-0.20	0.347
TSS	TSS	TSS	TSS	TSS	TSS	58 241 040	cg00677455	0.03	0.03		2.6	0.1	-0.01	0.838	0.09	0.677
TSS	TSS	TSS				58 241 201	cg02732509	0.21	0.22		0.0	0.0	0.02	0.7694	0.24	0.25
TSS	TSS	TSS				58 241 216	cg23105471	0.06	0.06		0.0	0.0	-0.09	0.0935	0.35	0.093

Mini-plots "*delta beta-value (Tumor minus Normal), paired samples*" show difference of beta-values (beta value is a ratio of methylated DNA in the sample at current position), between a tumor and adjacent normal tissue. Vertical axis scale is from −1 to +1. Red – hypermethylation, blue – hypo-methylation. HM score – hypermethylation score based either on comparison of beta-value distribution across two pools (tumors and norms) or on the pairwise comparison for matched tumors and normal tissues. Predicted chromatin states: E – enhancer, WE – weak enhancer, TSS – promoter region, PF – promoter flanking regions, CTCF – insulator element, R – repressed region, T – transcribed region.

Table S4. Top predicted microRNA regulators of genes *CTDSP1/2/L* and *RB1.*

	RB1		***CTDSP1***		***CTDSP2***		***CTDSPL***	
1	mir-106a	-0.21	mir-183	-0.46	mir-15a	-0.31	mir-18a	-0.51
2	mir-26a-2	-0.24	mir-182	-0.41	mir-34a	-0.19	mir-181b-1	-0.43
3	mir-335	-0.24	mir-501	-0.28	mir-16-1	-0.23	mir-16-2	-0.53
4	mir-221	-0.27	mir-500a	-0.31	mir-15b	-0.20	mir-181a-1	-0.33
5	mir-26b	-0.10	mir-324	-0.11	mir-150	-0.21	mir-15a	-0.55
6	mir-675	-0.12	mir-362	-0.27	mir-423	-0.17	mir-181b-2	-0.32
7	mir-199a-2	-0.22	mir-149	-0.20	mir-195	-0.08	mir-15b	-0.51
8	mir-199b	-0.23	mir-654	-0.12	mir-182	-0.05	mir-100	-0.10
9	mir-199a-1	-0.22	mir-214	-0.11	mir-330	-0.15	mir-34a	-0.38
10	mir-26a-1	-0.05	mir-891a	-0.38	mir-130b	-0.28	mir-122	-0.51
11	let-7g	-0.09	mir-500b	-0.16	mir-497	-0.01	mir-155	-0.70
12	mir-127	-0.24	mir-125b-1	-0.14	mir-1270-2	-0.28	mir-17	-0.41
13	mir-20b	-0.11	mir-200c	-0.35	mir-1270-1	-0.28	mir-181d	-0.14
14	let-7e	-0.07	mir-874	-0.35	mir-590	-0.25	mir-142	-0.53
15	mir-149	-0.26	mir-660	-0.26	mir-221	-0.10	mir-21	-0.68
16	mir-29a	-0.33	mir-22	-0.17	mir-185	-0.22	mir-25	-0.52
17	mir-30b	-0.46	mir-141	-0.32	mir-424	-0.03	mir-93	-0.56
18	mir-98	-0.04	mir-29b-2	-0.32	mir-196b	-0.05	mir-106b	-0.62
19	let-7d	-0.05	mir-532	-0.24	mir-625	-0.22	mir-195	-0.14
20	mir-218-2	-0.32	mir-20b	-0.30	mir-3613	-0.31	mir-885	-0.28

Top-20 potential regulating microRNA for *RB1* and *CTDSP1/2/L*. The values in each cell represent Spearman's correlation coefficient between the gene and the microRNA expression level. This is also represented with cell color. The bars inside cells indicate prediction score of microRNA binding site according to several databases (from left to right): miRTarBase (strong experimental evidence), TargetScan (conservative binding sites, CS), PicTar (CS), DIANA microT, mirSVR (CS), TargetScan (non-CS), PicTar (non-CS), miRTarBase (weak experimental evidence), mirSVR (non-CS). Cell borders indicate miRNA-gene pairs with predicted binding site (dashed border – only non-CS sites; double border – several reliable algorithms). MicroRNAs are sorted by overall prediction score (decreasing).

Table S5. Clinical characteristics of patients with ccRCC.

Age	Average 53<br>Range 32–80
Gender	Men 48% (25/52)<br>Women 52% (27/52)

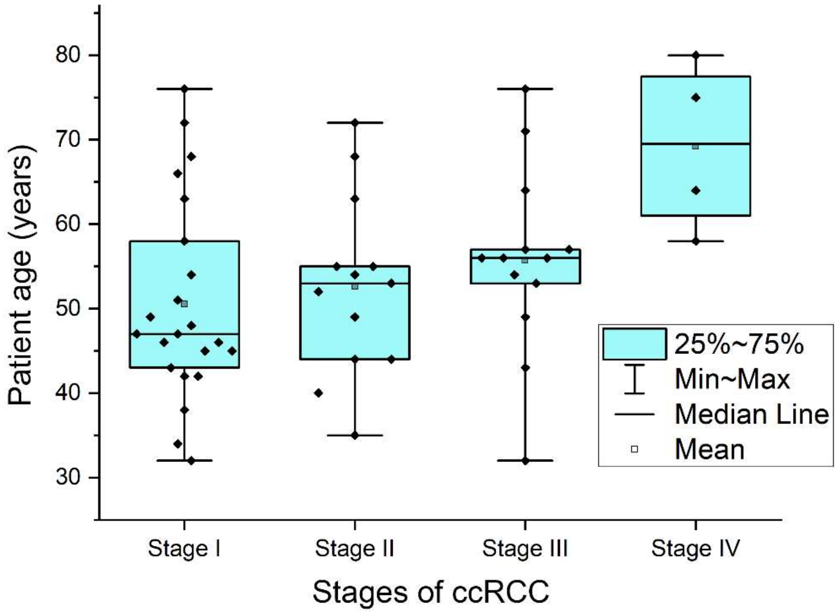

In total there were: 22 ccRCC stage I samples, 13 stage II samples, 17 stage III samples and 4 stage IV samples, which due to low statistical significance were not used in the calculations.

Supplementary Figure S1–S3

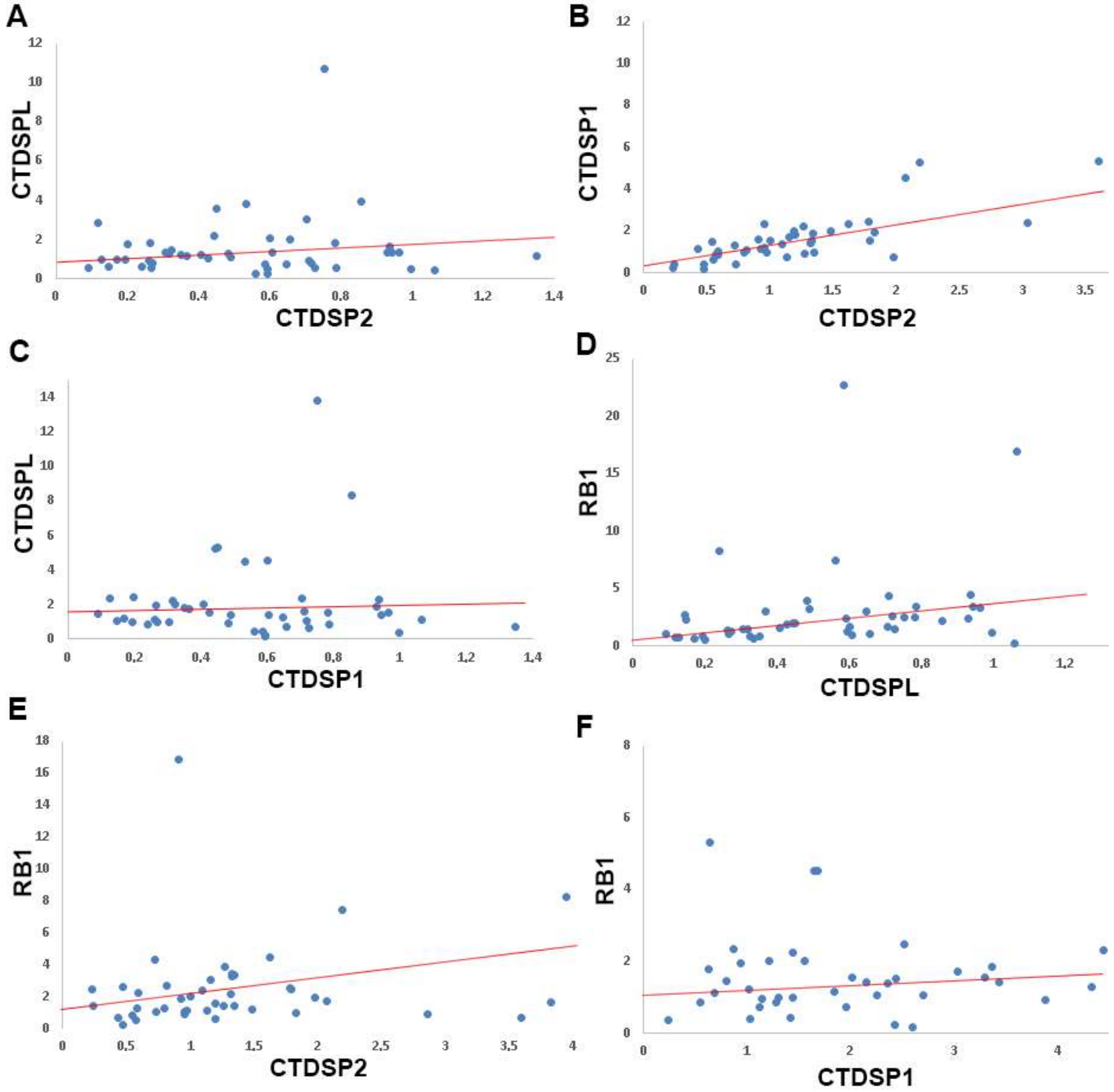

Figure S1. Results of the correlation analysis of the expression of the *CTDSP1*, *CTDSP2*, *CTDSPL* and *RB1* genes. The axes show the values of the relative expression levels. The correlation of expression for genes *CTDSPL* and *CTDSP2* (**A**), *CTDSP1* and *CTDSP*2 (**B**), *CTDSPL* and *CTDSP1* (**C**), *RB1* and *CTDSPL* (**D**), *RB1* and *CTDSP2* (**E**), *RB1* and *CTDSP1* (**F**) is presented. Statistically significant co-expression was revealed only for two pairs: *CTDSP1* and *CTDSP2* (r_s= 0.76; P < 0.001) (**B**); *CTDSPL* and *RB1* (r_s= 0.38; P < 0.05) (**D**). No significant differences were found in the case of male and female patients and in tumors with and without metastases (P > 0.05).

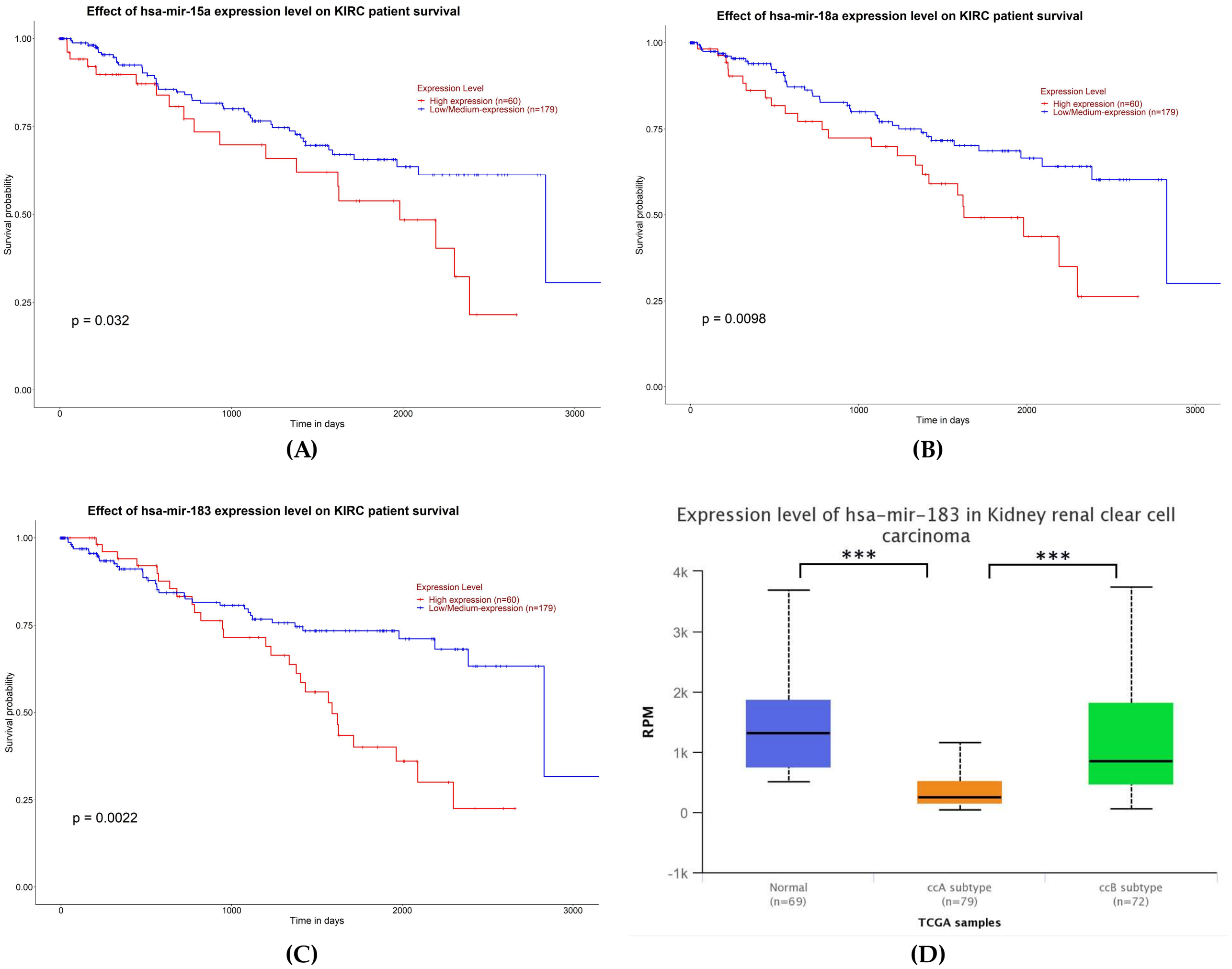

Figure S2. Survival analysis of microRNAs (predicted by CrossHub) using UALCAN web portal. Effect of mir-183 expression level on KIRC patient survival (**A**), effect of mir-15a expression level on KIRC patient survival (**B**), effect of mir-18a expression level on KIRC patient survival (**C**), expression of mir-183 in KIRC depending on ccRCC subtypes (**D**). *** $p < 0.001$

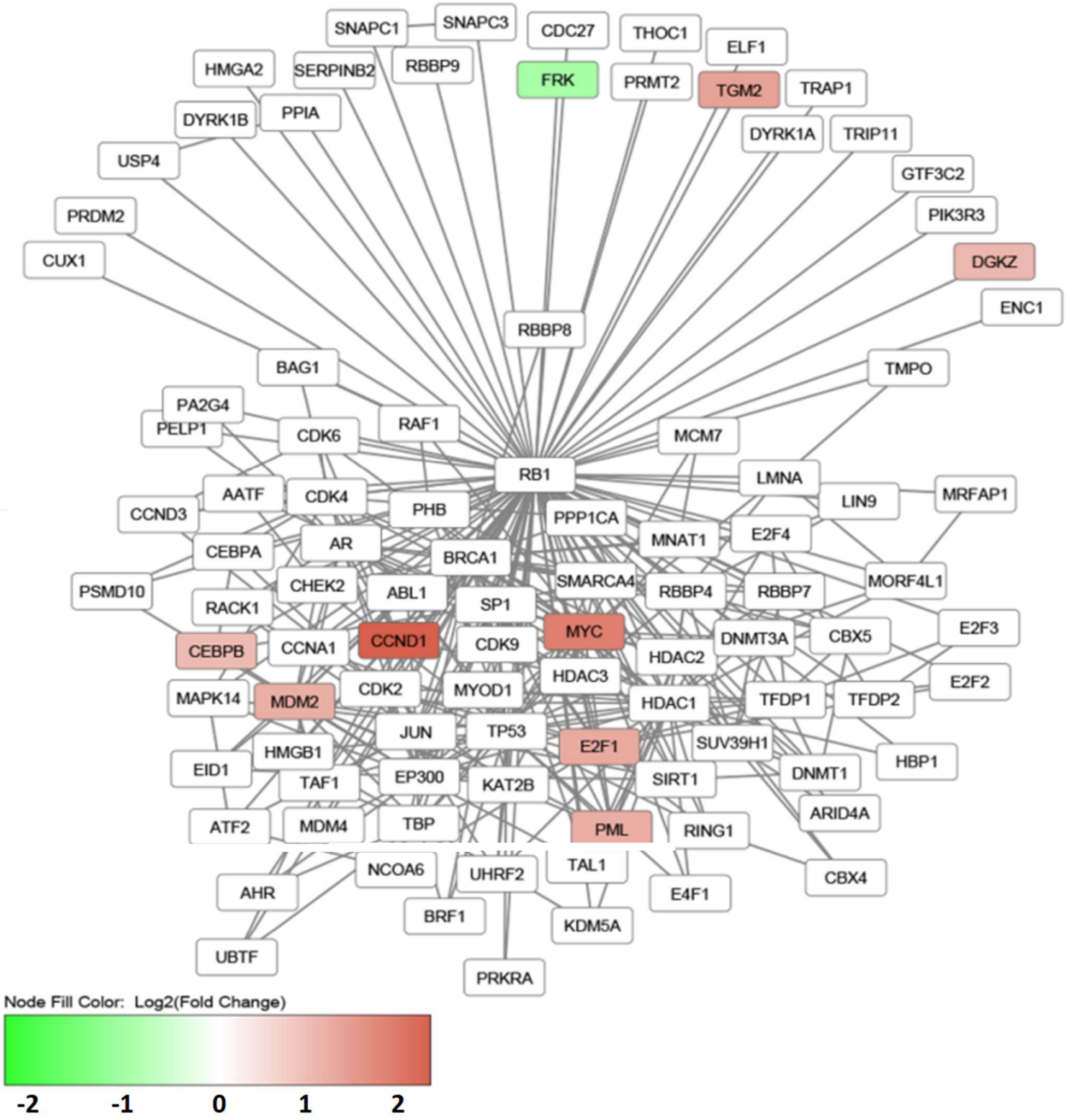

Figure S3. Protein-protein interactions networks (including the data from high-throughput screens) for *RB1* taking into account differentially expressed genes in ccRCC. The genes with decreased and increased expression are marked with a gradient from green to red (color scale represents log2 of expression level fold change, tumor versus normal). The network was inferred using the GPS-Prot (BioGrid data). Differentially expressed genes in ccRCC (TCGA; KIRC dataset) were derived with ANOVA algorithm using GEPIA2.
